# Editorial: A human perspective on robotic hand design, analysis, control and beyond

**DOI:** 10.3389/frobt.2026.1820835

**Published:** 2026-03-23

**Authors:** Kunpeng Yao, Yuri Gloumakov, Julia Starke

**Affiliations:** 1 School of Computer Science, University of Leeds, Leeds, United Kingdom; 2 Electrical and Computer Engineering Department, University of Connecticut, Storrs, CT, United States; 3 Institute of Robotics and Cognitive Systems, Section Computer Science/Technology, University of Lübeck, Lübeck, Germany

**Keywords:** grasping, hand prostheses, humanoid hand, learning from human demonstration, robot manipulation, semi-autonomous control

## Introduction

Robotic hands and upper-limb prostheses stand at the intersection of mechanical design, intelligent control, and human experience, representing one of the most challenging frontiers in robotics and human-machine interaction. Despite remarkable progress in actuation, sensing, and learning, achieving manipulation capabilities that are robust, adaptable, and intuitive remains a fundamental challenge. With their remarkable and efficient manipulation skills, the human hand can serve as an inspiration to further advance the development of robotic hands. Therefore, we believe that the human perspective is key to address the robotic challenges we are facing in manipulation. Robotic hands are inspired by, interact with, assist, or replace human hands, and must therefore be understood not only as technical systems, but also as embodied interfaces between humans and machines. The Research Topic, “*A Human Perspective on Robotic Hand Design, Analysis, Control and Beyond*” was conceived to highlight the research work that places the *human* at the center of robotic hand research: whether as user, teacher, or source of inspiration.

**FIGURE 1:**
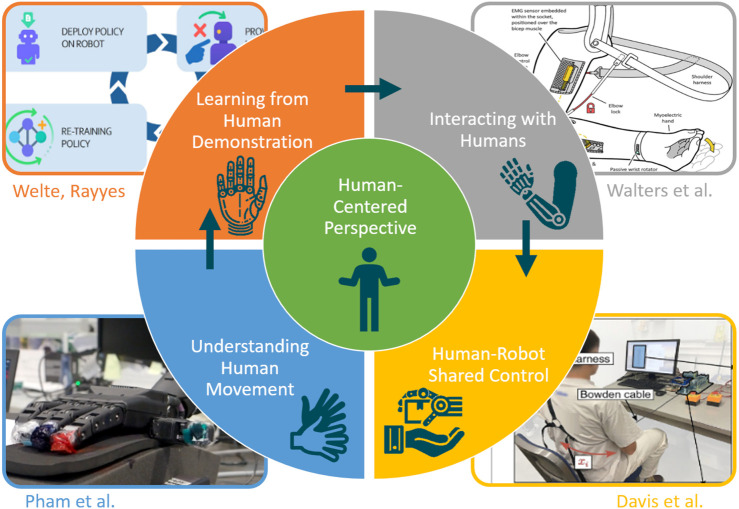
Human-centered robotic hand research: the human serves as teacher, partner and user to advance the grasp and manipulation capabilities of modern robotic hands; the small pictures in boxes illustrate the work presented by the publications in this Research Topic (from the original publications clockwise from lower left): Pham et al., Welte and Rayyes, Walters et al. and Davis et al. (all pictures are cropped and used under CC BY).

The four contributions selected in this Research Topic reflect this perspective by spanning learning paradigms, dexterous manipulation, clinical prosthetics, and human-centered robotic assistance, as depicted in Figure 1. Together, they illustrate how advances in robotic hands increasingly emerge from the integration of human factors with robust engineering and learning-based approaches.

## Article summaries

On the Research Topic of robot learning, Welte and Rayyes provide a comprehensive survey of interactive imitation learning for dexterous robotic manipulation. This?> work frames dexterous manipulation as a fundamentally human-centered learning problem, characterized by high-dimensional control, limited training data, and safety constraints. By synthesizing literature across imitation learning, reinforcement learning, and hybrid approaches, the authors argue that incorporating humans directly into the learning loop through real-time feedback and corrective guidance offers a promising path toward sample-efficient and robust manipulation skills. The survey reviews both current dexterous manipulation methods and interactive learning techniques from other robotic tasks, identifying a notable gap: while interactive learning has succeeded in simpler domains, its application to multi-fingered, contact-rich manipulation remains limited, outlining a key direction for future research.

In order to address the challenges of robotic hand control, Pham et al. learn from intuitive human manipulation strategies. Their work focuses on the representation and generalization of cleaning tasks, which are practically relevant yet underexplored. The authors introduce a framework based on motion synergies that is inspired by neuroscience and combines principal component analysis (PCA) with probabilistic movement primitives (ProMPs). By these means, the generalized human hand motions on the level of finger-joint kinematics can be transferred into robotic hand control. The work demonstrates accurate posture reconstruction and compositional generalization, allowing to adapt the demonstrated motions across different tasks. The synergy-based control is successfully deployed both in simulation and on real robotic hands. This work considers real-world manipulation motions beyond grasping and uses motion strategies learned from human demonstration as a powerful prior for learning reusable and interpretable manipulation skills.

The human perspective is equally central in prosthetics. Walters et al. contribute a scoping review that demystifies hybrid upper-limb prostheses, with a particular emphasis on hybrid-power systems that combine body-powered and externally powered components. Their analysis reveals a disconnect between *clinical practice*, where hybrid solutions are frequently prescribed, and the *academic literature*, where such systems remain poorly defined and underexplored. By proposing a clear taxonomy of hybridization and identifying key use cases, this review highlights the need for more systematic evaluation and standardized research into these devices. It also emphasizes the importance of aligning research agendas with the realities of clinical decision-making and user needs.

Complementing this structural perspective, Davis et al. examine how robotic assistance can be integrated into body-powered prosthetic hands without undermining the user’s sense of agency. Through controlled experiments with a prosthesis emulator, the authors show that assistance timed to align with natural human reaction latencies can simultaneously improve grasp performance and preserve subjective agency. Even though users are physically aware of the assistance through the device’s inherent force feedback, their sense of agency is not undermined. This work provides important empirical evidence that automation and human control need not be opposing forces. Instead, it demonstrates how careful temporal and perceptual alignment can enable assistive technologies that cooperate and augment, rather than replace, human intention.

Together, these papers highlight a unified research theme: progress in robotic hand design and control depends not only on making systems more powerful or more autonomous, but on making them more human-aware. Whether through interactive learning, synergy-based representations, clinically grounded prosthetic classifications, or agency-preserving assistance, each article emphasizes that understanding and respecting human behavior is essential for creating effective hardware, control and learning algorithms of robotic hands.

## Outlook

Looking forward, we believe that a truly human-centered approach will shape the next-generation of robotic hands and prosthetic devices. Future systems will increasingly blur the boundaries between human and machine, by learning from humans, adapting with humans, and acting in ways that feel natural and reliable to humans. Achieving this vision will require continued collaboration across robotics, neuroscience, health science, and human-computer interaction. We hope that this Research Topic not only captures the current state of the art on the Research Topic, but also inspires further work that places the human perspective at the center of robotic hand research.

